# Impact of Chikungunya Virus Infection on Health Status and Quality of Life: A Retrospective Cohort Study

**DOI:** 10.1371/journal.pone.0007800

**Published:** 2009-11-11

**Authors:** Man-Koumba Soumahoro, Patrick Gérardin, Pierre-Yves Boëlle, Joelle Perrau, Adrian Fianu, Jacques Pouchot, Denis Malvy, Antoine Flahault, François Favier, Thomas Hanslik

**Affiliations:** 1 Université Pierre et Marie Curie, Unité Mixte de Recherche en Santé 707, Paris, France; 2 Institut National de la Santé et de la Recherche Médicale, Unité de Recherche 707, Paris, France; 3 Centre d'Investigation Clinique - Épidémiologie Clinique (CIC – EC) de La Réunion (Institut National de la Santé et de la Recherche Médicale/Centre Hospitalier Régional/Union Régionale des Médecins Libéraux de La Réunion), Groupe Hospitalier Sud - Réunion, Saint Pierre, La Réunion, France; 4 Institut National de la Santé et de la Recherche Médicale, Unité Mixte de Recherche en Santé 953, IFR69 Recherche épidémiologique en santé périnatale et en santé des femmes et de l'enfant, Saint-Vincent de Paul, France; 5 Assistance Publique Hôpitaux de Paris, Service de Santé Publique, Hôpital Saint-Antoine, Paris, France; 6 Assistance Publique Hôpitaux de Paris, Service de Médecine Interne, Hôpital Européen Georges Pompidou, Paris, France; 7 Université Bordeaux 2, Service de Maladies Infectieuses et Tropicales, Hôpital Saint-André, Bordeaux, France; 8 École des Hautes Études en Santé Publique, Rennes, France; 9 Assistance Publique Hôpitaux de Paris, Service de Médecine Interne, Hôpital Ambroise Paré, Boulogne Billancourt, France; 10 Université Versailles Saint Quentin en Yvelines, Versailles, France; Washington University School of Medicine, United States of America

## Abstract

**Background:**

Persistent symptoms, mainly joint and muscular pain and depression, have been reported several months after Chikungunya virus (CHIKV) infection. Their frequency and their impact on quality of life have not been compared with those of an unexposed population. In the present study, we aimed to describe the frequency of prolonged clinical manifestations of CHIKV infection and to measure the impact on quality of life and health care consumption in comparison with that of an unexposed population, more than one year after infection.

**Methodology/Principal Findings:**

In a retrospective cohort study, 199 subjects who had serologically confirmed CHIKV infection (CHIK+) were compared with 199 sero-negative subjects (CHIK–) matched for age, gender and area of residence in La Réunion Island. Following an average time of 17 months from the acute phase of infection, participants were interviewed by telephone about current symptoms, medical consumption during the last 12 months and quality of life assessed by the 12-items Short-Form Health Survey (SF-12) scale. At the time of study, 112 (56%) CHIK+ persons reported they were fully recovered. CHIK+ complained more frequently than CHIK– of arthralgia (relative risk = 1.9; 95% confidence interval: 1.6–2.2), myalgia (1.9; 1.5–2.3), fatigue (2.3; 1.8–3), depression (2.5; 1.5–4.1) and hair loss (3.8; 1.9–7.6). There was no significant difference between CHIK+ and CHIK– subjects regarding medical consumption in the past year. The mean (SD) score of the SF-12 Physical Component Summary was 46.4 (10.8) in CHIK+ versus 49.1 (9.3) in CHIK– (p = 0.04). There was no significant difference between the two groups for the Mental Component Summary.

**Conclusions/Significance:**

More than one year following the acute phase of infection, CHIK+ subjects reported more disabilities than those who were CHIK–. These persistent disabilities, however, have no significant influence on medical consumption, and the impact on quality of life is moderate.

## Introduction

Chikungunya is a disease caused by an arboviral alphavirus transmitted to humans by *Aedes* mosquitoes (*Aedes aegypti*, *Aedes albopictus*). This disease is endemic to many countries in western, central, eastern and southern Africa, Indian Ocean and West Pacific Islands and South-East Asia [1]. Before 2005–2006, outbreaks of Chikungunya had never been described on the islands of Indian Ocean (Comoros, Mayotte, Madagascar, La Réunion, Mauritius and Seychelles). Since then, many imported cases due to this arbovirus were reported in areas where the disease is not endemic.

Chikungunya virus (CHIKV) is an enveloped, RNA positive-strand Alphavirus belonging to the Togaviridae family [2]. This virus can target human epithelial and endothelial cells, fibroblasts and macrophages [3] and muscle progenitor cells [4], to cause a wide range of clinical manifestations including fever, headache, rash, nausea, vomiting, myalgia, and, especially, disabling joint pain [5], [6]. CHIKV can damage collagen and alter connective tissue metabolism in cartilage and joints to produce severe acute arthritis. This is characterized by necrosis and fibrosis, as indicated by high levels of urinary proline, hydroxyproline and mucopolysaccharides observed in patients during the acute phase of the infection [7]. In skeletal muscles, atrophy and necrosis of scattered muscle fibres and vacuolization of cells were shown to be potential underlying mechanisms for a myositis syndrome [3]. Whether these phenomena can evolve towards degenerative chronic lesions remains to be demonstrated.

Nevertheless, several authors have reported persistent clinical manifestations (mainly joint and muscular pain and depression) several months after acute infection, leading some of them to consider the possibility of chronic forms of Chikungunya infection [5], [8]–[13]. These persisting symptoms are not consistent among patients and whether they are attributable to CHIKV or represent the exacerbations of underlying conditions is still questionable. The frequency of persisting symptoms has not been compared with that of an unexposed population without CHIKV infection. Moreover, the burden of such disabilities on quality of life is a challenging, previously unrecognized problem with potentially alarming economic consequences [14].

We conducted an investigation to describe the frequency of late clinical manifestations of Chikungunya virus infection and to measure the impact on quality of life and health care consumption, compared with an uninfected population, in the context of the epidemic occurring in La Réunion Island.

## Results

Among the 434 people contacted, 27 refused to respond to the questionnaire and 9 were excluded from the analysis because of the impossibility of matching, leaving 199 pairs for analysis. The participation rate was 92% (398 out of 434).

The mean age of the study population was 42 years (range: 2–91). Table 1 shows the characteristics of the study population. There were as many men as women, and participants were uniformly distributed by area of residence.

**Table 1:** Characteristics of the study population (199 matched pairs).

Sociodemographic characteristics	Number of people/total number of participants (%)
**Gender**	
Female	202/398 (51)
Male	196/398 (49)
**Age**	
Under 30	136/398 (34)
30 to 59	158/398 (40)
60 and over	104/398 (26)
**Residential area**	
North	94/398 (24)
South	104/398 (26)
East	100/398 (25)
West	100/398 (25)

**Figure 1:**
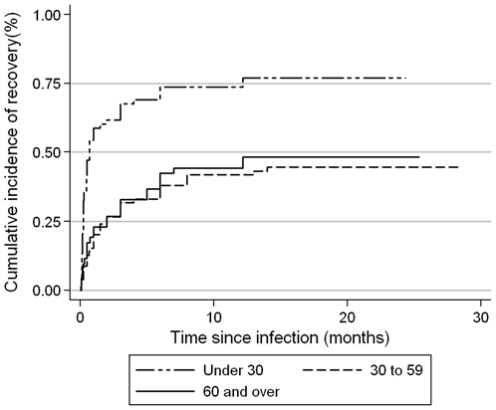
Percentage of patients (n = 199) fully recovered, shown by age group. The long dash followed by the short dash represents patients aged under 30; the dash represents patients aged 30 to 59 and the solid line represents those aged 60 and over. Recovery is fast in young subjects.

Following an average time of 17 months (range: 5–28) from the acute phase of disease, 112 of the 199 CHIK+ subjects (56%) reported that they were fully recovered. The CHIK+ subjects aged under 30 reported a faster recovery than the older subjects (75% versus 40% at one year; p<0.001) (Figure 1). There was no evidence of a difference in recovery rates between men and women (59% versus 54%; p = 0.42).

Symptoms at the time of study are shown in Table 2. CHIK+ subjects complained significantly more often of joint pain than CHIK– subjects (RR = 1.9 [95% CI: 1.6–2.2]). The attributable risk percent of CHIKV infection in CHIK+ subjects for arthralgia was 46.7% (95% CI: 37.3%–54.7%). According to topographical distribution, the pain was located in both upper and lower limbs, but less frequently involved the spine. CHIK+ cases also reported myalgia (RR = 1.9 [1.5–2.3]), fatigue (RR = 2.3 [1.8–3.0]), depression (RR = 2.5 [1.5–4.1]) and hair loss (RR = 3.8 [1.9–7.6]) more frequently than CHIK– subjects. The attributable risk percent of CHIKV in CHIK+ subjects was 46.4% (34.5%–56.2%) for myalgia, 56.3% (43.5%–66.3%) for fatigue, 60% (33.8%–75.8%) for depression and 73.7% (47.2%–86.9%) for hair loss. At the time of study, there was no difference between the two study groups regarding sleep disturbances and digestive or skin manifestations.

**Table 2:** Reported symptoms and medical consumption.

	CHIK+	CHIK–		
Symptoms	n (%)	n (%)	RR* [95% CI]	p-Value†
**Arthralgia**	105 (53)	56 (28)	1.9 [1.6–2.2]	<0.001
Upper limbs	76 (38)	29 (15)	2.6 [2.0–3.3]	<0.001
Lower limbs	83 (42)	33 (17)	2.5 [2.0–3.2]	<0.001
Spine	43 (22)	25 (13)	1.7 [1.2–2.3]	0.01
**Myalgia**	84 (42)	45 (23)	1.9 [1.5–2.3]	<0.001
**Fatigue**	71 (36)	31 (16)	2.3 [1.8–3.0]	<0.001
**Depression**	25 (13)	10 (5)	2.5 [1.5–4.1]	0.014
**Hair loss**	19 (10)	5 (3)	3.8 [1.9–7.6]	0.007
**Skin disorders**	20 (10)	10 (5)	2.0 [1.2–3.4]	0.087
**Sleep disorders**	55 (28)	39 (20)	1.4 [1.1–1.8]	0.076
**Digestive disorders**	12 (6)	11 (6)	1.1 [0.6–1.9]	1
**Medical consumption in past twelve months**				
Taking analgesic	52 (26)	45 (23)	1.2 [1.0–1.4]	0.42
Medical consultations	159 (80)	169 (85)	0.9 [0.9–1.0]	0.25
Hospitalization	14 (7)	18 (9)	0.8 [0.5–1.2]	0.57

CHIK+, persons with a history of CHIKV infection confirmed by serology (n = 199); CHIK–, persons confirmed as seronegative for CHIKV (n = 199); RR, relative risks; CI, confidence interval.

* RR, relative risks controlling for stratification criteria.

† Exact Mac Nemar test.

There was no significant difference between CHIK+ and CHIK– subjects in the frequency of use of analgesics, medical consultations or hospitalizations in the last 12 months (Table 2).

Quality of life was assessed in all subjects aged 15 or over (N = 324). The average score (SD) of the physical component summary (PCS) was significantly lower in CHIK+ subjects than CHIK– (46.4 (10.8) versus 49.1 (9.3)) (Table 3).

**Table 3:** Quality of life assessment by the SF-12 of subjects with a history of Chikungunya virus infection compared with uninfected subjects.

	CHIK+	CHIK–	
SF-12 component summaries	mean score (SD)	mean score (SD)	p-Value*
**Physical Component Summary**	46.4 (10.8)	49.1 (9.3)	0.04
**Age**			
Under 30	53.4 (5.1)	52.8 (6.0)	0.66†
30 to 59	46.0 (10.4)	50.2 (8.2)	0.02†
60 and over	42.5 (11.9)	45 (11.0)	0.35†
**Gender**			
Female	45 (11.2)	48.9 (9.6)	0.02†
Male	47.9 (10.0)	49.2 (9.0)	0.71†
**Mental Component Summary**	45.5 (11.1)	45.6 (10.1)	0.83
**Age**			
Under 30	46.4 (9.7)	46.1 (9.5)	0.97†
30 to 59	43.9 (11.7)	44.7 (9.5)	0.58†
60 and over	47.3 (10.9)	46.4 (11.4)	0.84†
**Gender**			
Female	43.8 (11.2)	43.9 (10.8)	0.81†
Male	47.5 (10.8)	47.2 (9.1)	0.91†

CHIK+, persons with a history of CHIKV infection confirmed by serology (n = 162); CHIK–, persons confirmed as seronegative for CHIKV (n = 162).

* Wilcoxon's rank test for paired samples.

† After Bonferroni correction, the statistical significance was then set at p = 0.01 with a bilateral formulation.

Compared with the CHIK– subjects, the CHIK+ subjects' worst score for the PCS was found in patients aged 30 to 59 and in females (Table 3). After Bonferroni correction, however, there was no significant difference according to age and gender. Regarding the mental component summary (MCS), there was no difference between CHIK+ and CHIK– subjects according to either age or gender.

## Discussion

Here we report a comparative study of longstanding disabilities owing to CHIKV infection as they are reported within the community. It showed that several subjective symptoms (arthralgia, myalgia, fatigue, depression, hair loss) reported by people infected by CHIKV more than one year after acute infection may be attributable largely (46.4 to 73.7%) to the past infection. The perceived quality of life in these subjects was barely changed, however, as demonstrated by the SF-12. In addition, CHIKV infection was shown to have no significant impact on the use of analgesics, medical consultations or hospitalizations.

To the best of our knowledge, no controlled survey had yet attempted to establish the accountability of an arbovirosis, both on the persistence of non-specific symptoms and on their impact on quality of life in a community. Existence of a “chronic form” of the disease following CHIKV infection had been suggested by uncontrolled observational studies previously reported [5], [11] although this study design did not allow non-specific symptoms to be attributed to the disease. The reported percentage of people with symptoms ranged from 48% to 93% six months following CHIKV infection [10], [11] and remained up to 64% 18 months post infection [5], [15]. In addition, 15% of subjects complained of persistent arthralgia 20 months after infection [8], while 12% complained of residual symptoms such as pain and/or stiffness more than three years later [16]. In these reports, the causal relationship of such symptoms with CHIKV infection is questionable owing to participation and selection biases, absence of standardization in symptom elicitation, lack of clinical review and the absence of a control group. Moreover, specificity of reported clinical manifestations may be limited. For example, the prevalence of self-reported musculoskeletal pain lasting for more than one week in the past month, including rheumatic symptoms, was 47% in an otherwise healthy adult population in the UK [17]. With such a large prevalence of rheumatic symptoms in the community, these symptoms are likely to occur frequently in subjects long after a Chikungunya virus infection even without a causal relationship.

Chronic symptoms following infection with other “arthritogenic” alphaviruses such as Ross River virus and Sindbis-related virus have been reported previously in series of case studies [13], [18]–[23]. Concurrently, following the reports of long-term clinical manifestations of Ross River virus disease, two prospective longitudinal studies were conducted, one of them based on clinical review [24], [25]. It was concluded that earlier studies may have overestimated the prevalence and duration of these symptoms, showing their progressive resolution within three to six months. Moreover, conditions such as chronic rheumatic disease or depression were identified in half the cohort. In a previous study reporting on the persistent arthralgia associated with CHIKV infection, 44% of patients reported a prior history of joint symptoms which made it difficult to establish the real increase in symptomatic functional manifestations [5]. Similarly, Sissoko et al. identified the presence of underlying osteoarticular comorbidity as a significant risk factor for non recovery [15]. That is why the possibility of overlap between a rheumatic pre-existing underlying condition and an authentic Chikungunya-related arthralgia led the most skeptical authors to think that prolonged signs of “arthritogenic” alphaviruses could represent the neglected mode of entry of a rheumatic disease, such as rheumatoid arthritis or psoriasic rheumatism [5], [19], [22], [24].

The basis for the musculoskeletal symptoms of Chikungunya disease is still poorly understood [26]. To the best of our knowledge, the presence of CHIKV in human synovial tissue has never been reported, although there is some evidence that CHIKV can damage the cartilage and has a tropism for the fibroblasts of joint capsule and skeletal muscle [7], [27]. One of the recurrent symptoms of the disease is myalgia. In patients with myositis syndrome, three to four months after the acute phase of the infection, viral antigens were found selectively inside skeletal muscle satellite cells, a type of cell responsible for postnatal muscle growth and repair [4]. It has been hypothesised that infection of these cells might have pathological consequences for long-term myalgia in infected patients. A recent animal model also demonstrated the presence of CHIKV antigens within young mice muscles several weeks following infection [28]. The pathogenic significance of these observations still needs to be clarified. As suggested by some authors, the muscle and joint connective tissues targeted by the virus contain high amounts of nociceptive nerve-endings that may account for the muscle and joint pain [27].

A post-infective fatigue syndrome similar to the symptoms reported here by CHIK+ patients (i.e. disabling fatigue, musculoskeletal pain, neurocognitive difficulties and mood disturbance) has been shown to persist for six months or more in 12% of persons following a variety of acute infections, including Ross River virus [29]. It seems to routinely occur at a similar incidence after each infection and to be predicted mostly by the severity of the acute phase of illness. Thus, the high frequency of such late symptoms following CHIKV infection could simply reflect the fact that the symptoms of the acute phase of CHIKV infection are usually more severe and incapacitating than other common viral infections [30]. The neurobiological mechanisms that could explain this post-infective fatigue syndrome remain to be determined [29].

Little attention was paid to scalp lesions in CHIKV infection. However, in the field of alphaviral infections, hair loss is consistent with alopecia encountered in a mice model of Chikungunya infection [28] and in one of O' Nyong - Nyong fever [31]. The basis for this symptom following Chikungunya infection is not understood. It could simply reflect a non specific symptom triggered by the acute febrile illness, as observed after many systemic diseases [32].

Quality of life was already assessed with the SF-12 in other rheumatic conditions, such as rheumatoid arthritis, osteoarthritis and fibromyalgia [33]–[35]. In these diseases, patients usually had an MCS above 40, as observed in CHIKV-infected individuals. The PCS of rheumatological patients, however, frequently lay around 30, which is a much lower (i.e. poorer) score than that observed among Chikungunya cases. The relatively well preserved quality of life of CHIK+ patients is illustrated by the fact that more than a year after the onset of infection, Chikungunya-associated symptoms were not associated with any increase in health care consumption. This is a reassuring finding, although we cannot exclude the possibility that CHIK+ patients engaged in higher use of alternative medicines, such as herbal medicines [36]. It is also likely that this finding reflects an adaptive coping strategy to manage chronic symptoms without additional medical consumption.

There is no clear explanation why patients under the age of 30 reported faster recovery compared to those older than 30. Older age and severe initial joint pain have been recognized as risk factors for non-recovery [15], and older patients can experience more severe acute infections than younger patients. Indeed, inpatients with acute infections during the La Réunion outbreak were older than outpatients (64 versus 48 years respectively, p = 0.001) [6]. Finally, underlying medical conditions such as osteoarthritis, whose prevalence increases with age, could also result in delayed recovery [15].

Our report has some strengths and limitations. The investigator was not blinded to the serological status of the subjects; participants were questioned about symptoms long after the acute phase of the disease. Also patients were not examined so alternative diagnoses could not be excluded. However, in contrast with previous studies, a standardized health status questionnaire was used and administered by only one investigator, a validated generic quality of health assessment tool was used (the SF-12), a control group was included for comparison and participants were randomly selected through a population-based cohort [37]. There was no substantial participation bias favouring patients with more severe illness, whereas previous studies had included patients who presented severe forms of CHIKV infection, often requiring hospitalization, and who were thus not representative of the general population. The diagnostic accuracy of CHIKV infection was efficient, as it has been reported that during La Réunion's epidemic the incidence of asymptomatic cases (i.e. all subjects who were not aware of their infection during the epidemic period but were tested serologically positive to CHIKV) and the rate of false positives (i.e. subjects who declared that they were infected during the epidemic period but had negative serology) were lower than 5% [10], [37].

Whilst there is appreciable temporary disability associated with acute CHIKV infection, and even though more frequent manifestations are reported by infected persons more than a year after the acute phase of the condition, the resulting impact on quality of life and medical consumption seems moderate. Despite alarming signs observed throughout the outbreak in La Réunion, such as reports of severe atypical forms[38], [39], mother-to-child intra partum transmission [40] and increased mortality [41], these results reintroduce Chikungunya in the field of “benign” illnesses. Further research, most appropriately population-based studies using clinical reviews, is required to better understand the natural history, pathogenesis and long-term impact of persisting manifestations following CHIKV infection.

## Materials and Methods

### Study design and population

We conducted a retrospective cohort study to compare subjects who had been infected by CHIKV during the 2005 and 2006 outbreaks in La Réunion Island with those who had not been infected. The study sample was derived from the SEROCHIK survey, a cross-sectional, population-based seroprevalence study aimed at assessing the prevalence of CHIKV infection in the community soon after the 2006 outbreak [37]. This survey involved a representative sample of 2442 people for whom the history of Chikungunya infection was recorded and a serological test performed using an ELISA assay [42]. In this survey, the sensitivity of CHIKV specific-IgG serology was 97.9% (95% CI: 88.7–99.9%) and the specificity 100% (96.0–100%) [37].

From this population, we selected pairs of subjects with one subject with serologically confirmed CHIKV infection (CHIK+) and one matched subject with negative serology (CHIK–). Pairs of subjects were obtained by balanced random sampling without replacement from 24 strata defined by a unique combination of age (<30 years old, 30 to 59 years and ≥60 years), gender and area of residence (north, south, east and west of the island area).

### Setting

La Réunion is a French overseas department of 787,836 inhabitants located on a volcanic island of 2511 km2 belonging to the Mascarene Islands in the south-western Indian Ocean. Between 2005 and 2006, a Chikungunya outbreak occurred in La Réunion, in which 38.2% of inhabitants became infected [37].

### Data collection

Participants were interviewed by telephone between March and June 2007. The questionnaire was administered by one investigator (MKS), using closed questions addressing current symptoms, medical consumption during the last 12 months (consultation, hospitalization and use of analgesics) and quality of life assessed by the SF-12 scale [43].

The patients were questioned about the presence or absence of symptoms on the day of the telephone interview. Illness history was recorded during the interview using questions formulated in lay terms. They were asked if they were currently experiencing the following: joint pain (for arthralgia), muscle aches (for myalgia), fatigue, depression, hair loss, skin disorders, sleep disorders or digestive disorders such as nausea, vomiting or diarrhea. When patients reported presence of arthralgia, they were asked to localize the involved joints.

The SF-12 explores the physical and mental aspects of quality of life [17]. The physical component summary (PCS) assesses physical functioning difficulties caused by physical problems, bodily pain and general health. The mental component summary (MCS) assesses vitality, social functioning and role functioning difficulties caused by emotional problems and mental health. The PCS and MCS are standardized to reflect a general population mean of 50 and a standard deviation of 10. Higher scores indicate better functioning [44].

For children under eight, the questionnaire was completed by parents. The SF-12 scale addresses only people of 16 years and over.

### Sample size and statistical analysis

We considered that an absolute difference of 15% in the frequency of musculoskeletal (e.g. arthralgia or myalgia) symptoms between CHIK+ and CHIK– subjects would be of interest. It has been reported that about 45% of the adult population suffer musculoskeletal pain [18] and the sample size calculation for a 5% type 1 error and a 20% type 2 error yielded 186 subjects in each group. For practical reasons, we finally decided to include 400 subjects (200 CHIK+ and 200 CHIK–).

Assessment of explanatory variables was conducted by univariate analysis. For paired samples, qualitative variables were analysed using exact Mac Nemar's test and continuous variables (SF-12 components) were analysed using Wilcoxon's rank test. Adjusted relative risks (RR) and their 95% confidence interval (95% CI) were calculated for each persisting symptom controlling for stratification criteria (age, gender, and area of residence) using Mantel-Haenszel methods [45]. Attributable risk percents (ARP%) and their 95% CI were calculated for each significant persisting symptom to assess the accountability of CHIKV infection, as follows: ARP% = (RR−1)/RR×100. We used the Kaplan-Meier method to estimate the time to full recovery in CHIK+ patients and the factors influencing the recovery time. Statistical significance was set at p = 0.05 with a bilateral formulation.

For the evaluation of quality of life, specific analyses were conducted separately in groups based on sex and age. We then performed five tests adjusted by Bonferroni correction and the statistical significance was set at p = 0.01 with a bilateral formulation.

EpiData 3.1™ software (EpiData Association, Copenhagen, 2003) was used for data entry and Stata 10.0™ software (StataCorp. 2008, Texas, USA) for analysis.

### Ethical considerations

During the SEROCHIK survey, which had received ethical approval, the participating subjects had been informed that they might be called back for ancillary research. During the telephone interview, the objectives of the study were presented and oral consent to participate was obtained. For people under the age of 18, the inform consent form was completed by parents.
